# The Principles of Art Therapy in Virtual Reality

**DOI:** 10.3389/fpsyg.2018.02082

**Published:** 2018-10-31

**Authors:** Irit Hacmun, Dafna Regev, Roy Salomon

**Affiliations:** ^1^Faculty of Social Welfare and Health Sciences, School of Creative Art Therapies, University of Haifa, Haifa, Israel; ^2^Gonda Multidisciplinary Brain Research Center, Bar-Ilan University, Ramat Gan, Israel

**Keywords:** virtual reality, art therapy, presence in immersive virtual environments, digital art, perspective-taking

## Abstract

In recent years, the field of virtual reality (VR) has shown tremendous advancements and is utilized in entertainment, scientific research, social networks, artistic creation, as well as numerous approaches to employ VR for psychotherapy. While the use of VR in psychotherapy has been widely discussed, little attention has been given to the potential of this new medium for art therapy. Artistic expression in VR is a novel medium which offers unique possibilities, extending beyond classical expressive art mediums. Creation in VR includes options such as three-dimensional painting, an immersive creative experience, dynamic scaling, and embodied expression. In this perspective paper, we present the potentials and challenges of VR for art therapy and outline basic principles for its implementation. We focus on the novel qualities offered by this creative medium (the virtual environment, virtual materials, and unreal characteristics) and on the core aspects of VR (such as presence, immersivity, point of view, and perspective) for the practice of art therapy.

## Introduction

Virtual reality (VR)^1^, allows an interactive experience within a simulated, computer generated environment. Current VR systems, in which participants typically wear a head mounted display (HMD), allow audio-visual sensory simulation of realistic or fictional environments with increasing fidelity and realism. VR has recently become a promising tool for scientific investigation (Bohil et al., 2011) and is now used extensively to study behavioral (Banakou and Slater, 2014; Debarba et al., 2017) and neural processing (Ionta et al., 2014; Herbelin et al., 2015; Limanowski et al., 2017). The capacity to simulate different realities and experiences have also prompted the use of VR in psychotherapy where VR techniques have been implemented in the treatment of phobias, PTSD, and anxiety disorders (Rothbaum et al., 2002; Parsons and Rizzo, 2008; Beidel et al., 2017), depression (Falconer et al., 2016), schizophrenia, eating disorders (Gutiérrez-Maldonado et al., 2016), and pain management (Freeman et al., 2017). Recent technological advances have allowed the proliferation of VR technologies from specialist laboratories to widespread consumer applications, increasing the availability of such systems, and enhancing the possibilities of their use for therapy (Bohil et al., 2011) as well as entertainment and art (Bates, 1992; Carrozzino and Bergamasco, 2010; Gates et al., 2011).

Beyond scientific and clinical applications, VR has also created a novel medium for artistic expression (e.g., Google Tilt brush, Oculus quill, Oculus medium, Blocks) allowing unique and unfamiliar forms of creativity and extending classical forms of expression such as painting and sculpturing. In this perspective paper, we explore how this novel artistic medium provided by VR can be employed for clinical purposes under the framework of art therapy.

## Virtual reality as an artistic creative medium

Art making is an innate human tendency. It has been argued that along with speech and tool making, this activity could be used to define our species (Dissanayake, 2001). Indeed, artistic mediums such as painting and sculpting have been a fundamental form of human expression since prehistoric times (Bégouen, 1929). Throughout history, technological developments have influenced and changed artistic expression (Benjamin, 2010). As such, the evolution of digital technology, boosted by the introduction of personal computers, has generated new forms of art such as digital painting, image and video editing, and multimedia art (Wands, 2007). Today, creation with digital art tools are not merely restricted to the use of professional artists, on the contrary, its high accessibility and friendly user interface, has made it a common form of expression. Here, we will focus on artistic creation in VR using currently available commercially art software for VR (e.g., Tilt Brush, Google, Palo Alto), to demonstrate the characteristics of this new medium and suggest their possible potential for the use in art therapy.

## Characteristics of the virtual creative medium

### VR creative environment

**Figure 1:**
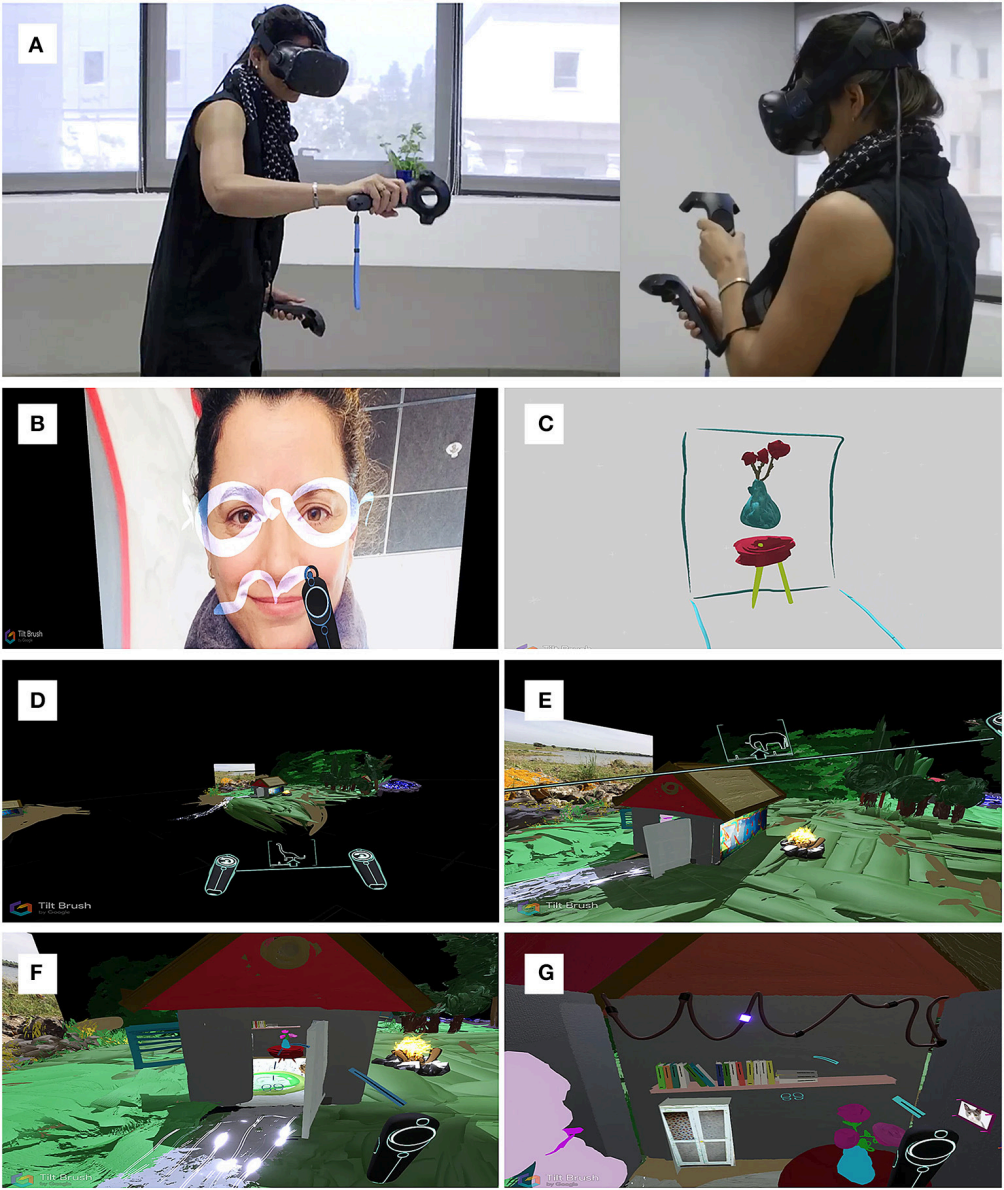
Artistic creation in VR. **(A)** The virtual reality setup. The creator wears an HMD and creates via the hand-held controllers, see also Supplementary Videos. **(B)** Photographic stimuli from the real world can be introduced as backgrounds or objects in VR, allowing interactions between real and virtual worlds. **(C)** Creation in VR includes unreal characteristics such as absence of gravity in a 3D environment. **(D,E)** The VR environment allows dynamic rescaling of the virtual world. The creator can modify the size of the artistic creation (and by proxy their relative size) at will. **(F,G)** As the virtual creation is a scalable 3D environment, the creator can “step into” his creations, enhancing the immersivity, and scope of the artistic creation.

Creation in VR combines elements from the world of painting (line, patch, shape, color, 2D), elements from the world of sculpting (3D), and novel elements enabled by the digital medium. This unique combination is on one hand similar to the classical mediums (painting, drawing, sculpturing) but on the other hand, fundamentally different. The VR creative environment includes a VR system (e.g., Oculus rift, HTC Vive) and an enclosed space of about 2 m^2^ for motion. The creator wears an HMD and holds a wireless controller in each hand. The controllers are used for artistic creation, with one controller for painting, and one for the color palette and interface menu (resembling the classical medium of brush and color plate, Figure 1A). The creator can move around freely, in the immersive three-dimensional space, while highlighted grids in the visual display appear when approaching the boundaries of the physical room. The environment's visual background can be easily controlled and changed, and the creator can choose from a variety of backgrounds, ranging from a single color to a customized chosen photo or scenery (Figure 1B, Video S1).

The creation itself involves hand and/or full body movements stimulating an embodied experience. This encourages the artist to extend his expression from the typical finite canvas in front of her, to form boundless environments in 360° around her (Video S2). Furthermore, different perspectives, are possible for both the creator and external observer watching the creative environment on a computer display. For example, the observer has the option to choose his perspective, whether he would like to see from the creator's first-person perspective (1PP) or set an exterior fixed perspective (third person perspective-3PP) (Video S3). Upcoming technological developments will enable integrating more than one person in the VR environment allowing shared creation.

### Virtual material

Contrary to classical mediums such as painting and sculpting the materials employed here are themselves, virtual. As such VR painting has no substance and no tactile feedback, neither from the material nor from the canvas, similarly, to painting with a computer mouse, spray-painting, or drip painting. Note however, that haptic feedback in VR is rapidly becoming feasible (Popescu et al., 2000). The creation with the virtual material is similar to painting, but unlike painting, the creator can paint in 3D. Thus, it offers the possibility to view the creation from more than one angle as when viewing a sculpture. Moreover, there is the unique possibility, due to the virtuality of the materials, to “step” inside or through elements in the creation (Figures 1F,G, Video S4). When the artist wishes to apply a change or correction to the artwork, the medium offers full flexibility with actions such as erasing, undo, and/or redo (Video S5). As for storage of the creative “product,” it can be saved at any time as a digital format, moreover it can also be stored as a video format which will enable tracking of any point in time during the creative process (see Table 1 for summary of differences).

**Table 1:** Comparison of VR art therapy and classical Art therapy mediums.

	**Classical mediums**	**Virtual reality**
**CLINICAL SETTING**		
Therapist client physical interaction	Client and art therapist in direct contact	Client and art therapist in virtual contact

Eye contact	Yes	No
Perspective	Third person	First person/Third person
Facial expressions	Visible	Partially obscured
Technological requirements	Low	High
**CREATIVE EXPERIENCE**		
Material	Physical	Virtual
Artistic product	Physical	Digital
Dimensionality	2D/3D	2D/3D/4D
Immersivity	Low	High
Realism (laws of physics)	Bounded	Unbounded
Size of creation	Fixed as chosen	Unbounded
Haptic feedback	High	None
Possibility of tele-therapy	Low	High
Sense of privacy/isolation	Low	High

### Un-real characteristics

A novel aspect of creation in VR is that it allows expression which is unrestricted by natural physical laws. For example, 3D objects can be suspended in midair seemingly defying gravity (Figure 1C, Video S6). Furthermore, it is possible to create elements whose properties (e.g., color, location) dynamically change over time (Video S6). The color palette includes besides the natural colors also a wide range of unrealistic and fantastic colors (Video S6). An innovative and unique attribute of VR art is the dynamic control of spatial dimensions. The canvas size is practically infinite, and the creator can re-scale and change the size of his creation along the creative process (Figures 1D,E, Video S7). Additionally, it is possible to create multisensory relations such as adding music which may modify aspects of the artistic creation (e.g., colors). Thus, artistic creation in VR builds on classical aspects of artistic creation (e.g., color palette, brushes), while augmenting these with novel features which are unique to VR (e.g., scalability, dynamic objects).

## Potentials of VR for art therapy

A key aspect of VR for psychotherapy is the ability to induce a feeling of “presence” in the computer-generated world experienced by the user (Riva et al., 2007, 2016). By mimicking the sensory (i.e., visual, auditory) and motor (e.g., immersive environment, motion tracking) signals and contingencies found in the real world, VR allows the creation a subjective experience giving the individual illusion that the experience is real (Riva, 1998). This sense of presence can be a powerful therapeutic tool promoting personal change and self-reflectiveness, as it offers the individual the opportunity to “experience” (Riva et al., 2016). Moreover, VR is often referred to as an “advanced imaginal system:” an experiential form of imagery that is highly effective in inducing emotional responses (Vincelli, 1999; Vincelli et al., 2001; Riva et al., 2016). The specific implementation of VR in psychotherapy depends upon the psychological approach used and is customized to the specific disorder and patient being treated. Multiple techniques employing simulative controlled exposure (e.g., anxiety, phobias, fear of flying), embodied technologies (e.g., eating disorder), cue exposure (e.g., addictions), or distraction (e.g., pain management) have been explored (for review see Riva, 2005). This, specialization however is costly as it requires to develop multiple environments adapted to the specific disorder and patient (e.g., EMMA, Alcañiz et al., 2007).

Art therapy is a form of psychotherapy which employs artistic creation for integrative personality processes (Guttmann and Regev, 2004). Art therapy typically consists of an interaction of an individual or group with a therapist who supports self-expression through various artistic mediums. It has been suggested that such artistic expression, in itself (Kramer and Wilson, 1979; Rubin, 2016) or accompanied by verbal reflection (Naumburg, 1953; Dalley and Case, 2014) is effective in raising psychological well-being and treatment of clinical syndromes.

Digital arts, as new mediums of creation have produced new forms of expression for art therapy (see various examples in Garner, 2016). The unique characteristics of VR experience, compounded by the novel possibilities of artistic expression in VR further expand these therapeutic possibilities (Brown and Garner, 2016; Lohrius and Malchiodi, 2018).

Moreover, when using art therapy in VR, clients create their own customized environment in the processes of therapy. Thus, circumventing a limitation of previous approaches to VR based psychotherapy. We suggest that VR based therapy, combining individualized creative processes in the unrestricted VR environment forms a therapeutic environment which can be well-tailored to the clinical needs of each individual.

### Presence and immersivity

VR allows to immerse the participant in a virtual environment, creating a sense of *Presence* defined as the illusion of “being there” (Minsky, 1980; Sanchez-Vives and Slater, 2005). The sense of presence is suggested to be grounded in the embodied and interactive control afforded by real time sensorimotor correlations similar to those underlying the sense of bodily self in the real world (Sanchez-Vives and Slater, 2005; Slater et al., 2008; Blanke, 2012; Salomon, 2017). Indeed, research in cognitive neuroscience has shown that such sensorimotor and multisensory contingencies are the foundation of the sense of self in the world (Ehrsson, 2007; Slater et al., 2008; Blanke, 2012; Salomon et al., 2013; Hara et al., 2015) and thus their implementation in VR allows Presence in the virtual world. For example, the use of HMDs obscuring vision of the real world, allowing 360° fields of view and tracking the head movements of the participant promote the sense of Presence (Slater and Sanchez-Vives, 2016). The sense of presence coupled with modifiable environments in VR allow transformations of the sense of self (Slater et al., 2010; Rognini et al., 2013; Herbelin et al., 2015). For example, when sensorimotor correlations between the self and virtual body of a child are introduced, changes in perception and implicit attitudes were found, causing a shift toward experiencing the world as a child (Banakou et al., 2013). Critically, in VR art therapy this active sensorimotor engagement can enhance Presence within the artistic creation itself and may potentially lead to an augmented experience of artistic creation compared to non-immersive situations.

### Point of view and perspective

Another interesting potential for art therapy in VR relates to the possibility of taking different visual perspectives. In the VR medium, the therapist has the option to choose between observing the clients' creation process in the VR setting from their natural perspective (3PP) or from the client's 1PP (Video S3). This possibility of experiencing the clients' artistic creation in 1PP during therapy is novel and may have an interesting clinical potential. Empirical and theoretical studies have shown that the cognitive and neural processing of perspective are tightly linked to empathy and mentalization, underpinning humans' ability to assess mental representations as well as affective states of other individuals (Decety and Jackson, 2004; Lamm et al., 2007; Corradi-Dell'Acqua et al., 2008; Schnell et al., 2011). For example, imaging visual perspectives (e.g., 1PP or 3PP) may affect mentalization (Langdon and Coltheart, 2001; Frith and Frith, 2006) or empathy (Lamm et al., 2007). VR allows actual manipulations of subjective viewpoint (Slater et al., 2010; Debarba et al., 2017) which, often employed together with visuo-motor correspondences, can induce changes in stereotyped thinking (Yee and Bailenson, 2006), interpersonal attitudes (Peck et al., 2013), or cognitive and physiological processing (Banakou et al., 2013; Bergouignan et al., 2014). Thus, changes in perspective may cause substantial shifts in perceptual, social, and cognitive processing which may have valuable clinical implications (Libby et al., 2009).

In order to understand the possible impact of this feature on therapist and client, we must consider the role of perspective in theoretical conceptualizations of psychotherapy. Humanistic theories in psychology emphasize the importance of perceiving the client's inner world through his own personal perspective (Schneider et al., 2014). Carl Rogers, the founder of the client-centered therapy, suggested that the best vantage point for understanding behavior is from the perspective of the individual (Rogers, 1951). The option of perspective shifts in VR is also available to the client, who can decide if to view his creation from a 1PP or external 3PP viewpoint. Similarly, to the putative effects on the therapist's side, changing viewpoint may have considerable effects on the client as well, as such perspective shifts have been shown to affect motivation (Vasquez and Buehler, 2007), self-compassion (Neff, 2003), and have been employed in clinical settings (Gestalt empty chair technique) (Perls et al., 1951) and VR counseling (Osimo et al., 2015). Considering the potential of perspective shifts for modifying mentalization and empathy mentioned above, the possibilities of such shifts in VR art therapy may add novel and intriguing qualities in clinical practice.

### The potential virtual space

The virtual environment has been suggested to enable a synthesis of the actual and the imaginary (Vincelli, 1999). Through integration of unrealistic elements with an embodied, immersive sensory experience, it creates a dream like experience in a protected and controlled environment (Leclaire, 2003), (Video S8). This is reminiscent of Winnicott's suggestion of the “*potential space*” as an intermediate area of experiencing that lies between the inner world “inner psychic reality” (fantasy) and “actual or external reality” (Ogden, 2014). Winnicott states that: “It is a space where we can develop psychologically, to integrate love and hate and to create, destroy and re-create ourselves,” thus promoting the development of the self and facilitating psychological growth (Winnicott, 1997, p. 41). Indeed, the VR creative medium for art therapy may offer the creator a unique space in between fantasy and reality (Video S8), while being creative and playful, setting the ground for a conductive environment for therapy. Furthermore, the severance of the client from the real world through the immersion afforded by the HMD generates a sense of privacy and disentanglement from the external world. This private, dreamlike space incorporated within an immersive and enactive environment shows great potential for enhancing the efficacy of art therapy.

## Summary

In this opinion paper we presented the potentials of Art therapy in VR, focusing on fundamental aspects of this novel creative medium for clinical practice (Table 1). Several important aspects were not covered here but deserve mention. First, there is good reason to believe that the VR medium would appeal to younger generations which are highly engaged in the digital world possibly enhancing efficacy and compliance of treatments within these age groups (Bryanton et al., 2006). Furthermore, as VR systems become commercially available tele-treatment in art therapy is becoming a viable and feasible prospect (Collie and Cubranić, 1999). Finally, VR is enjoying a technological renaissance with novel solutions constantly emerging. Thus, several current technological constraints such as haptic feedback, monitoring of facial gestures, and group interactions are likely to be solved in the near future.

Despite the considerable potentials described above one must also consider the limitations and challenges of art therapy in VR. For example, while wearing a HMD there is no possibility for direct eye contact between therapist and client nor the ability to view facial expressions, fundamental aspects of human communication, interaction, and mentalization (Khalid et al., 2016; Ellis and Beattie, 2017). Furthermore, some people suffer from nausea and fatigue when using VR systems (cybersickness). The material art product is also different than classical art therapy mediums. While screenshots, videos, and even 3D images (e.g., Poly) or 3D printed objects of the artistic work can be generated these will not capture the full environment and scope of the artwork. However, this environment is digitally retained and can be revisited and continued along sessions.

Finally, the potency of VR is such that the creation in VR might have an overflow potential for some clients. The infiniteness of the virtual “canvas,” the immersivity and dynamic environments can have a powerful effect on the client, and to prevent overflow, the therapist must consider the suitability and extent of the VR medium to the client's needs.

In summary, we propose that art therapy is particularly suited for VR therapy as the clients themselves create the therapeutic environment that suits their specific needs. Furthermore, for art therapy the integration and implementation of new creative digital mediums in practice is crucial for the evolution of the field, and to best treat younger generations for whom which digital technology is an integrative part of their everyday lives. As technologies have driven novel forms of artistic expression and therapeutic possibilities (McNiff, 1999; Lynn Kapitan, 2007), we believe that VR has the potential to augment and enhance classical art therapy approaches.

## Author contributions

IH, DR, and RS envisioned the paper. IH and RS wrote the paper. IH, DR, and RS edited and finalized the paper.

### Conflict of interest statement

The authors declare that the research was conducted in the absence of any commercial or financial relationships that could be construed as a potential conflict of interest. The reviewer NC and handling editor declared their shared affiliation at the time of the review.

## Footnotes

^1^In this paper we use the general term VR to refer to immersive virtual reality in which a Head Mounted display is used to create an immersive experience.

## Supplementary material

Video S1 Variations of virtual reality backgrounds.

Video S2 Art creation engaging full body movements.

Video S3 First person perspective (1pp) <– –> Third person perspective (3PP).

Video S4 Step inside your creation.

Video S5 Flexibility of the creative medium.

Video S6 Un-real characteristics.

Video S7 Dynamic spatial rescaling in VR.

Video S8 Virtual potential space / Dream like state.
